# How to square the circle? A conceptual framework synergising strategies for circular agriculture to tackle climate change and enhance overall on-farm sustainability

**DOI:** 10.1007/s13280-025-02154-4

**Published:** 2025-04-10

**Authors:** Sinéad O‘Keeffe, Sophie Stein, Michael Curran, Lukas Baumgart, Sabine Zikeli, Marianna Siegmund-Schultze

**Affiliations:** 1 Agrosystems Research Group, Wageningen University & Research, 6700AA Wageningen, The Netherlands; 2 University of Hohenheim, Fruwirthstr. 14-16, 70599 Stuttgart, Germany; 3 Center for Organic Farming, University of Hohenheim, Fruwirthstr. 14-16, 70599 Stuttgart, Germany; 4 Leibniz Institute of Vegetable and Ornamental Crops, Theodor-Echtermeyer-Weg 1, Großbeeren, 14979, Germany; 5 Research Institute of Organic Agriculture (FiBL), Ackerstrasse 113, 5070 Frick, Switzerland

**Keywords:** Circular agriculture, Circularity strategies, Climate change strategies, Farm scale, Sustainability

## Abstract

There is an urgent need to change the current extractive and resource-intensive agricultural practices. Adopting circular practices within the agricultural system could provide multiple benefits of slowing global climate change, reducing extractive practices and helping farmers to adapt to a changing climate. However, there are still many barriers for farmers to adopt these desired circular agriculture (CA) practices, among others, a lack of information about on-farm circular practices. There is a need to support farmers in recognising which strategies can increase the circularity of their farm and what this means in terms of their farms’ climate neutrality and its long-term sustainability. Therefore, the aim of this paper is to develop a novel conceptual framework to facilitate a broader and integrated understanding of how on-farm CA strategies and practices contribute to the goals of climate change mitigation and on-farm sustainability, thus supporting farmers in transitioning their farms towards greater circularity.

**Supplementary Information:**

The online version contains supplementary material available at 10.1007/s13280-025-02154-4.

## Introduction

There is an urgent need to change the current extractive and resource-intensive agricultural production model (Dahlberg 1994; Bianchi et al. 2020; Sundkvist et al. 2005; Anderson and Rivera-Ferre 2021). Approximately one-third of the earth’s net primary production is used for food, feed, fibre, timber and energy, which impacts more than 70% of the earth’s ice-free land surface (IPCC 2019). Furthermore, the agriculture, land use and forestry sector is the second largest contributor to global greenhouse gas (GHG) emissions and it is predicted to increase in the coming decade (OECD et al. 2020; Crippa et al. 2021). A large share of these emissions relates to embodied emissions derived from fossil-based inputs, such as diesel, fertilisers, pesticides, along with land use change (Crippa et al. 2021; Lynch et al. 2021). Adopting circular practices within the agricultural and food sectors could help to reduce dependency on fossil-based inputs, as well as mitigate the need to extract additional raw materials and further agricultural expansion. In other words, circularity promotes doing more with less and, if implemented correctly, will ensure a reduction in GHG emissions and greater food security. Reducing the extractive nature of agriculture without compromising on food security is increasingly important, if we are to feed a projected population of approx. 9 billion people by 2050 (FAO 2009; van Dijk et al. 2021). Furthermore, such practices could provide the dual benefits of slowing global climate change and helping farmers to adapt to the impacts already occurring due to climate change in the Anthropocene (CGR 2021, 2022; Grumbine et al. 2021; Garré et al. 2022).

**Fig. 1:**
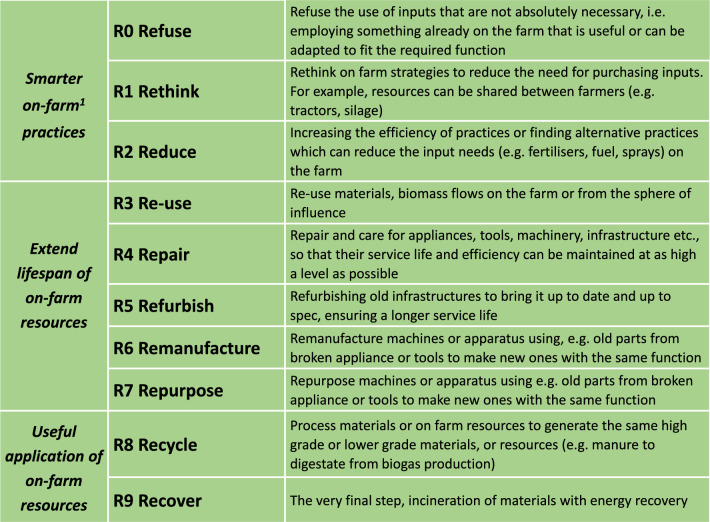
The 9R strategies for circularity, adapted from Potting et al. (2017) to the context of an on-farm situation (on-farm also refers to the sphere of influence of the farm, see Sect. “Implementation scale in a systems thinking context”)

Indeed, many global regions are pursuing the idea of circular agriculture (CA) in their attempt to reduce their emissions and meet their nationally determined contributions (NDCs).^1^ The European Union, for example, has outlined its approach for sustainable circular carbon cycles under the EU Green Deal (European Commission 2021). This covers all aspects of phasing out fossil-based carbon and phasing in renewable and biobased carbon sources. A key element of this approach is carbon farming (Euoropean Commission 2021), as this is seen to play a very important role in the EU meeting its climate change targets.^2^ The goals of these policies are to increase the climate neutrality of agricultural systems, through various mechanisms, including financial incentives for different farming approaches such as agroforestry, grasslands, peatland management and maintaining soil organic carbon in mineral soils. Many of the measures considered under these policies are also relevant for CA. Furthermore, to ensure synergies in climate change actions with biodiversity conservation (e.g. co-benefits), the EU’s Nature Restoration Law (NRL)^3^ will also play a role in determining which CA practices are feasible. How all of these policies align under the EU Member State Strategic Plans (2023–2027) for the Common Agri-cultural Policy (CAP) will be crucial for the promotion of CA on the ground (i.e. for farmer implementation). One example of an ambitious country-level policy comes from the Netherlands, which aims for greater sustainable agriculture through the circularity strategies of closing loops and regenerative practices to enhance the capacity of the land through nature-based interventions aimed at balancing food production, enhancing biodiversity and reducing waste (LNV 2019; Schut et al. 2021). This set of policies illustrates a comprehensive approach to phasing out fossil-based carbon while phasing in renewable and biobased carbon sources (e.g. 9R circularity strategies, Fig. 1).

However, there are still many barriers for farmers to adopt these desired circular practices. These relate to a lack of knowledge on circular practices, appropriate business models, higher level of risk associated with transitioning to CA, limited available labour, peer pressure to stick to current practices and unsuitable policies and legislation (Crovella et al. 2021; de Lauwere et al. 2022; Happel et al. 2022; López-Serrano et al. 2022; Rótolo et al. 2022). In their study investigating the barriers and drivers for adoption of CA, Bianchi et al. (2020) observed that “seeing is believing”, in other words when farmers could see CA practices being implemented successfully by other farmers, they were more likely to adopt them too. They suggested that pioneer farmers could play a role in encouraging and supporting other farmers and help to increase the diffusion of CA practices across the food system, a suggestion also supported by Silvius et al. (2023).

Indeed, de Lauwere et al. (2022) found, in their study of farmers implementing CA practices that, there was a strong and widespread desire to share knowledge among farmers. Furthermore, they identified that there is a need to support farmers in recognising which strategies are available for increasing the circularity of their farm and what this could mean for their farms’ climate neutrality and long-term sustainability goals. Therefore, to support this effort, the aim of this paper is to develop a conceptual framework to facilitate a broader and integrated understanding of how on-farm CA strategies and practices contribute to the goals of climate change mitigation and on-farm sustainability.

## What is circular agriculture?—Key definitions and characteristics

With an increasing interest in CA, there has also been an increase in scientific literature and reviews published in recent years (Velasco-Muñoz et al. 2021; Batlles-delaFuente et al. 2022). As a result of this, there are many definitions, descriptions, and goals for how the CA paradigm could look like (Table 1). Many of which encompass different aspects of the “R strategies” framework (Fig. 1). This is one of the most prevalent circular economy frameworks, and it has over time expanded from 3Rs—reduce, reuse and recycle––to the 9Rs, as shown in Fig. 1.

**Table 1:** Sample overview of definitions and descriptions for circular agriculture available in the literature (available at time of writing)

“Circular economy (CE) envisions food and agricultural systems that are coupled with material and energy flows at various levels of the value chain (production, processing, distribution, consumption) so that they can be reused as agricultural inputs (e.g. manure, composts, or sludges as biofertilisers; treated municipal wastewater as irrigation; food waste as animal feed), used in producing other valuable products (e.g. bioplastics and advanced biomaterials), or serve as sinks for waste (e.g. sequestration of atmospheric CO_2_ in soils). All of the above stems from the need to restore soil function and improve biodiversity in the farm and the local environment”	Basso et al. (2021)
“CE in reference to agriculture can be defined as the set of activities designed to not only ensure economic, environmental and social sustainability in agriculture through practices that pursue the efficient and effective use of resources in all phases of the value chain, but also guarantee the regeneration of biodiversity in agro-ecosystems and the surrounding ecosystems”	Velasco-Muñoz et al. (2021)
“Circular Agriculture is an ecological concept based on the principle of optimising the use of all biomass. Circular agriculture aims at closing the loop of materials and substances and reducing both resource use and discharges into the environment. It requires the development of more robust agro-ecosystems, which have an inherent capacity to maintain soil functions, to deal with pests, diseases and weeds, as well as with unfavourable weather conditions”	Bianchi et al. (2020)
“Circular agriculture seeks to close the life cycle of products, services, waste, water, and energy in order to seek better use and a reduction of the ecological impact”	Batlles-delaFuente et al. (2022)
“Circular agricultural systems involve: 1) system thinking to design closed cycles of nitrogen, phosphorus, carbon, energy and water along ecological cycles and waste treatment re-use along social value chains; 2) consideration of multiple organisms including microbes (bacteria and fungi), plants, animals, and insects as they form food webs from producers to decomposers; 3) innovations using smart design, digital technology, artificial intelligence, and big data; 4) and efficient and effective design and decision making across multiple scales throughout the entire value chain.”	Grumbine et al. (2021)
“Circular agriculture focuses on using minimal amounts of external inputs, closing nutrients loops, regenerating soils, and minimising the impact on the environment.”	Helgason et al. (2021)
“Circular agriculture is the optimisation of farm returns in the broad sense by using (their) own resources as much as possible and with respect for the environment (soil, air, water and nature quality, landscape value, climate and animal welfare)”	Erisman and Verhoeven (2019)

According to Kirchherr et al. (2017) a “*variety of understandings can result in (circular)…concepts eventually collapsing or ending up in conceptual deadlock*”. Therefore, a clear understanding of the key concepts and characteristics of CA is important to identify the potential benefits or burdens of adopting different circular strategies or activities. In several instances CA has been derived by adapting the circular economy concepts to agricultural systems (Ellen MacArthur Foundation 2015; Bianchi et al. 2020; Velasco-Muñoz et al. 2021; Batlles-delaFuente et al. 2022) (Table 1). Others were derived with a goal orientation focusing on the broader concept of sustainable food systems, i.e. not only agriculture but also value chains. For the latter, priorities have been set for circularity practices, with the understanding that food biomass produced on farms should be consumed by humans first and by-products still fit for animal consumption should be recycled back into the food system. In such CA systems animals should be used as vectors for this recycling, while producing high-quality food as a desirable co-product (de Boer and van Ittersum 2018; Van Zanten et al. 2019).

Additionally, there are CA concepts that originate with a broader focus on the concepts of the bioeconomy which also promotes the use of biomass to largely replace fossil-based production systems (e.g. bioplastics, energy). This concept in its most circular form (e.g. using the 9 Rs, Fig. 1) complements CA, as the bioeconomy seeks to explore options for the valorisation of agricultural wastes without creating issues of food and nutritional security (Bos and Broeze 2020; Velasco-Muñoz et al. 2021; Batlles-delaFuente et al. 2022; Khanna et al. 2024).

Descriptions for CA also refer to strategies, such as: narrowing (reducing) input streams, closing loops, slowing resource use and regenerating practices (Velasco-Muñoz et al. 2021). Others refer to diverse types of agricultural systems where CA contributes to increased sustainability, such as: multi-functional landscapes, sustainable intensification––focusing on nitrogen and crop-livestock management, digital agriculture and smallholder farmers (Grumbine et al. 2021). Most CA definitions and descriptions refer to the need to optimise, improve and reuse material, water and energy flows at various scales (e.g. farm, local ecosystems, value chains) to minimise the impacts of food production on the agro-ecosystem and environment, while enhancing the productivity of the farm (see Table 1).

In their review of the literature on CA, Yang et al. (2022) found a strong focus on natural science-based solutions, which in many cases neglected the social and political challenges required for adopting different circular management practices, particularly on the farm. They also found that there was a lack of focus on participatory approaches, which they argued to be a limitation of such technical definitions of CA. This is because many circular models are dependent on farmers’ adoption of CA practices and there is a strong need to have adequate legislation and policies for such models to be successful (e.g. the reverse cycling of wastes). Indeed, several authors have acknowledged that transforming agricultural systems into more circular ones requires more than just technological innovation; it also requires a cultural shift to establish more legitimate and long-term circular strategies (Dagevos and Lauwere 2021; Grumbine et al. 2021; Yang et al. 2022).

In distilling and compiling the descriptions and definitions, it can be seen that there are certain characteristics, conditions and ways of working that are required for achieving CA. These include, but are not limited to:

- A systems thinking approach—using multilevel perspectives to determine the most effective scale to work on in a very interconnected food system (i.e. nested agro-ecosystems, explained in the next section).
- CA practices need to tackle climate change through strategies, such as mitigation of GHGs, sequestration of carbon and carbon storage.
- CA practices may not necessarily be sustainable; therefore, considerations need to be made to ensure that CA activities not only tackle resource and pollution issues (e.g. mineral scarcity, climate change), but also synergistically contribute to multi-dimensional issues relating to sustainability (e.g. economic, social and environmental).
- CA practices need to have respect for nature and people, in terms of their needs, values and ethical considerations.
- CA practices implemented must ensure effective and efficient use of resources, using the 9Rs principles (Fig. 1) and aim to narrow and close the loop of materials and substances as far as possible to reduce resource consumption.
- CA is more than technological innovation; it is a multi-stakeholder process, involving farmers at its core.

Recognising these key characteristics is crucial when aiming to establish a conceptual framework to support and enhance the communication and actions of farmers in their transition towards enhanced sustainability through CA practices.

## Development of the conceptual framework

Currently there is no conceptual framework available for CA which not only focuses on technical aspects (e.g. nutrient, biomass and water cycling), but also includes other elements that contribute to climate change strategies and the multi-dimensional sustainability of the farm (e.g. social, agro-ecological).

In order to develop such a conceptual framework that could encompass the key characters of CA as outlined above, we needed to contextualise, build off and integrate four different concepts previously outlined in the literature in relation to circular economy, CA and climate change.

The very comprehensive circular economy framework of Elia et al. (2017) was developed to determine how well the circular economy paradigm has been adopted or implemented. They identified four key elements to include in their framework when monitoring for circularity. Adapted here to an agricultural context, these are:

1. The implementation scale (Sect. “Implementation scale in a systems thinking context”),
2. Strategies implemented (Sect. “Strategies to guide circular practices”),
3. Practices or aspects to monitor (Sect. “Aspects to monitor (AtM)”) and
4. Processes and flows to consider (Sect. “Processes and flows relevant for CA at farm level”).

Using the circularity framework of Elia et al. (2017) as our overarching framework, we take farm scale as the *implementation scale*, as farmers are at the core of CA practices. We integrate the four CA *strategies* outlined by Velasco-Muñoz et al. (2021) with the 9R circular *strategies* of Potting et al. (2017) to help characterise CA strategies implemented on the farm. Additionally, we link these CA strategies to strategies for tackling climate change. Furthermore, within the CA strategies, further *aspects to monitor* (AtM) are specified using the key performance criteria of Erisman and Verhoeven (2019). For the last element, *processes and flows to consider*, we extend the framework of Elia et al. (2017) using a life cycle thinking approach. We explain the different parts of the framework in more detail in the succeeding sections. The 9R strategies are outlined in Fig. 1, with examples provided.

### Implementation scale in a systems thinking context

In line with system thinking, agriculture and indeed CA can be considered as a nested system (Mang et al. 2016; Freeman et al. 2022a). This means that agriculture is not only a part of and contributes to a bigger system, such as the food system or the bioeconomy, but that it itself consists of smaller sub-systems, such as crop production or animal husbandry. Many times, these nested systems interact or influence one another; this is because current farming systems produce food, feed, fodder and materials for local, regional national, and global scales (Fresco et al. 2017; Freeman et al. 2022a). Furthermore, the management of agricultural resources and related supply chains are made up of a diverse set of social actors at various scales all with a broad spectrum of beliefs and interests (Yang et al. 2022). Therefore, boundary setting and determining the appropriate scale which can help to identify the benefits of circular strategies is not such an easy task with such nested and interacting systems (FAO 2014; Mang et al. 2016; Freeman et al. 2022a).

**Fig. 2:**
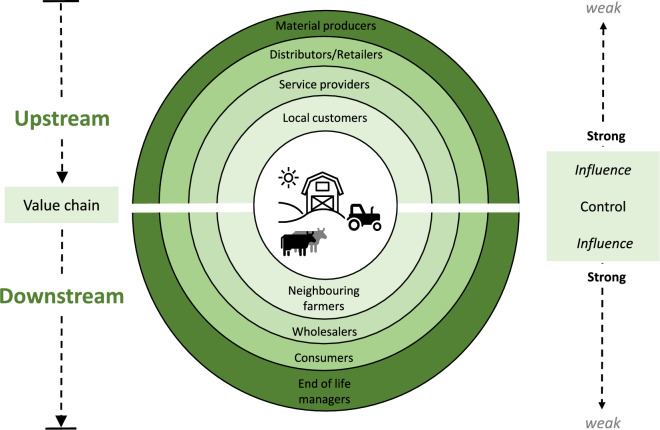
Graphical representation of potential scales and spheres of influence related to a farm, in the centre (white). The farmer at the centre has direct control over the practices and activities that happen on the farm; their influence over the practices of others declines the further you go away from the farm either upstream or downstream. Adapted from SAFA guidelines by FAO (2014)

At its core, based on the analysis in Sect. “What is circular agriculture?–key definitions and characteristics”, CA should be an approach that is people and nature centric which promotes the development of more robust agro-ecosystems and communities dependent on them. Therefore, it is crucial to recognise who will be the drivers of CA on the ‘‘ground” (CGR 2021). Who are the critical stakeholders? In the case of CA, it will fundamentally be farmers, landowners and the communities dependent on them. Indeed, the farm scale is one of the most important levers for enhancing the sustainability of agricultural and food systems, as many of the decisions which have serious environmental or social impacts occur at the farm scale (Foley et al. 2011; Schader et al. 2016). Therefore, the most basic unit to observe or anchor-targeted CA practices makes most sense at the farm scale and its associated potential spheres of influence (Fig. 2). Here, we refer to the spheres of influence as outlined in the SAFA (sustainability assessment of food and agricultural systems) guidelines (FAO 2014). What this means in relation to the context of CA, is that there is also a need to include, to a feasible extent, all enterprises exchanging products or services with the farm, in other words the extent to which the farmer has influence and may have greater opportunities to create change (Fig. 2). Understanding this can help farmers to support a systems thinking approach and help them to better manage their emissions and resource use (flows) on, in and off the farm, as effectively as possible within a geographically fixed area.

It is also crucial to understand the potential sub-systems (processes) within the farm boundaries, in order to start identifying the on-farm options farmers have to make their practices more circular, climate and nature friendly, while also contributing to sustainability. Such examples of on-farm practices and how they are circular are provided in Table 2 (linked to the 9Rs).

**Table 2:**
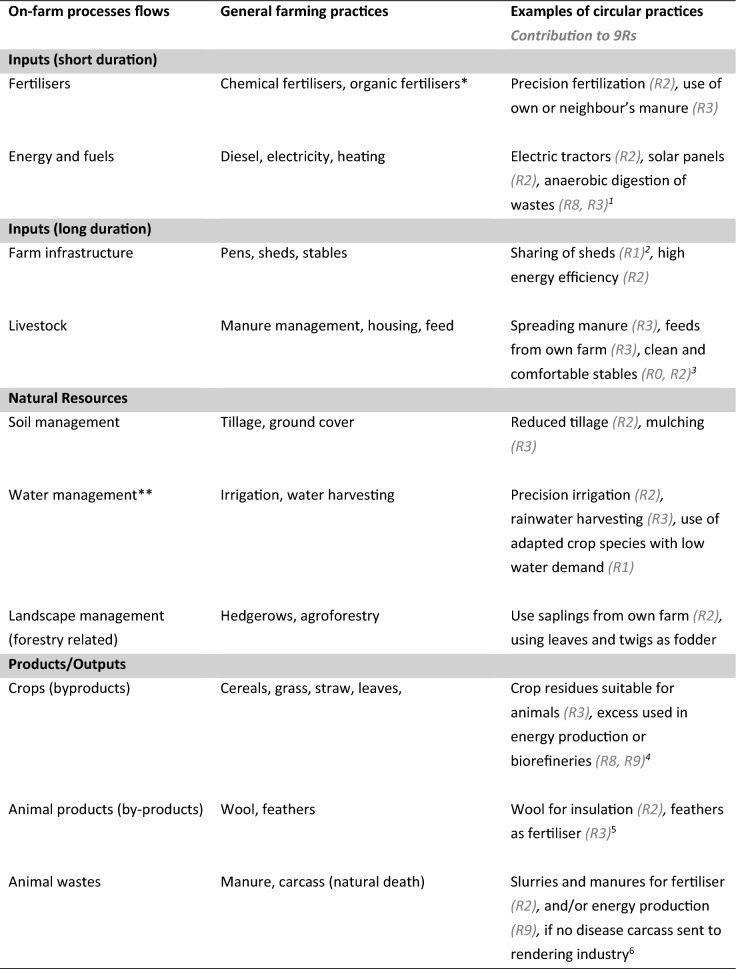
Examples for on-farm process and flows, coupled with practices which can be adopted to be more circular. Table derived from literature sources and authors’ collective knowledge (see table footnote)

^1^The activity of using an anaerobic digester (AD) or biogas to digest waste can be considered R9, but the use of the remaining digestate (fertiliser) after the AD process as fertiliser is considered R3

^2^Here we assume that farmers have combined efforts, a farmers collective to co-share sheds or infrastructures for their livestock (R1, rethinking)

^3^We assume here that the farmer takes high priority in the health and welfare of their livestock, as this has been shown to reduce GHG emissions and also reduce the need for higher replacements (Ali 2021; Hoffman and Valencak 2020)

^4^The use of straw and grass for biobased applications, such as insulation materials or as a source of sugars for plastic production (O’ Keeffe et al. 2011; Gomex-Campos et al. 2023;)

^5^Which R strategy used here depends on how the materials are processed and where the resulting product is used

^6^On the farm, manure can be managed either using an R8 strategy (e.g. as fertiliser) or an R9 (biogas -energy) strategy, but in relation to carcasses of healthy animals which are sent off farm to, for example, a rendering company, these can fall under a number of R strategies (e.g. R7- petfood, gelatine, R8, ingredients for soap), but these fall outside the scope of the farm and enter the bigger expanse of the food system, therefore, on the food system circularity level (Heinrich-Böll-Stiftung, Friends of the Earth Europe, BUND 2021)

References: (Bijttebier et al. 2014; Franzel et al. 2014; Pronk et al. 2015; Balafoutis et al. 2017; Hijbeek et al. 2019; Erisman and Verhoeven 2019; Araújo et al. 2021; Hercher-Pasteur et al. 2021; Pingoud et al. (2006); Sato and Nojiri (2019); Tagarakis et al. 2021; United Nations 2021; de Lauwere et al. 2022; Misbah et al. 2022; Velasco-Muñoz et al. 2022; Freeman et al. 2022b)

^*^While the application of organic fertilisers may be short term in relation to recycling them on the farm—the effects (e.g. soil organic carbon build up) are more long term

^**^Good soil management also leads to good soil–water management

### Strategies to guide circular practices

In their review of the literature, Velasco-Muñoz et al. (2021) identified four CA strategies. The first, narrowing resource loops, relates to using eco-efficient solutions to reduce the resource-intensity, environmental impact and resource leakage from the system (e.g. loss of nutrients through leaching). The second strategy, closing resource loops, follows the principles of cascading. This is an approach where a resource flow maintains its integrity and material usefulness for as long as possible, after which it will be sequentially reused for different purposes with decreasing usability until it will finally be used for energy production (Höglmeier et al. 2017). For example, a timber beam used as a structural component in a building, which is then used as a stake for a fence or other on-farm purpose and after some time finally used for firewood. The third strategy is slowing resource use and refers to increasing the longevity and lifespan of capital investments. The final strategy is regeneration, and this relates to all actions to enhance the social and natural capital of the farming region (e.g. farming community, soils, water, habitats). However, these strategies need to be broken down further into aspects that can make it easier to monitor and understand the CA performance of a farm; we explain these aspects in Sect. “Aspects to monitor (AtM)”. To ease the understanding of how the CA strategies of Velasco-Muñoz et al. (2021) can be implemented in practice on the farm, we link them with the 9R circular strategie*s* outlined in Fig. 1 and explained in more detail in the succeeding sections.

From Sect. “What is circular agriculture?–key definitions and characteristics” it was identified that one of the fundamental requirements of CA is to tackle climate change. Therefore, in addition to the integrated CA strategies outlined above, we link these CA strategies further to climate change mitigation strategies. There are three major strategies in which CA can contribute to tackling climate change. These are: (1) circularity for mitigation (Mit)^4^—through reduction or through substituting for a lower emitting product or approach, e.g. keeping fossil resources in the ground or preventing emissions from taking place in the first place, such as N_2_O emissions from soil; (2) circularity for sequestration (Seq)—removing existing carbon from the atmosphere (e.g. in soils, in living biomass such as—trees, grasslands); and (3) circularity for storage (Stor) in long-lasting products, the (biogenic) carbon pool products, such as timber or Miscanthus^5^ (Garcia-Chavez et al. 2022). The three strategies are not mutually exclusive and implementing one strategy may directly or indirectly result in the other strategy. In Table 3, we provide concrete examples of how circular practices on the farm (from Table 2) contribute to the three climate mitigation strategies. Furthermore, we also link these circular practices to potential co-benefits, in this way, creating a more concrete and holistic understanding of how CA strategies can be used to tackle climate change and improve overall on-farm sustainability.

**Table 3:**
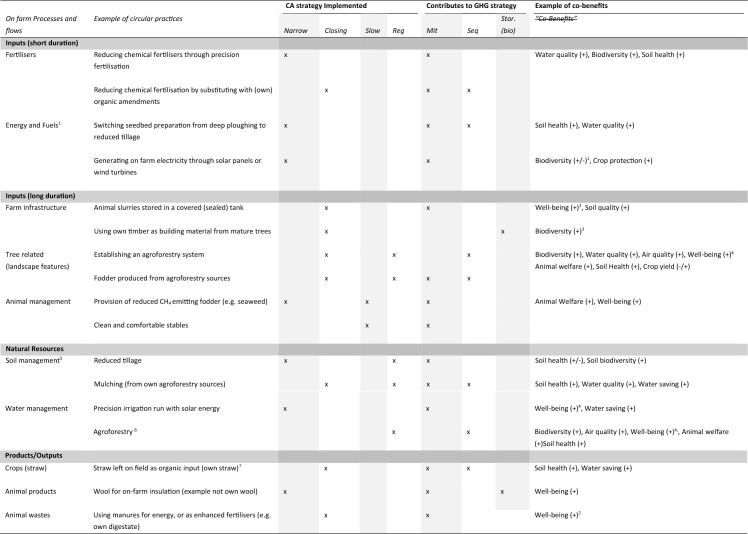
The on-farm CA practices outlined in Table 2 are used in this table to provide an overview of their related circularity strategy and strategies for tackling climate change, as well as their potential to create “co-benefits” on the farm for nature restoration and well-being

Narrow, narrowing loops; Closing, closing loops; Slow, slowing resource use; Reg, regenerative practices; Mit, mitigation; Seq, sequestration; Stor(bio), storage of biogenic carbon

^1^The effects of the solar panels can be positive or negative; it depends on the context of where the solar panels are situated. In dry regions, research suggests that solar panels can offer shade cover for plants and opportunities for pollinators in dry seasons (Graham et al. 2021).

^2^Well-being here refers to increased quality of life (and health) due to reduced emissions and odours and improved relations between the farmer and their neighbours. In the case of energy production from wastes, this refers to basic material for a good life (Millennium Ecosystem 2005).

^3^With proper biodiversity enhancing management practices in place (Ruokamo et al. 2023).

^4^Well-being here covers a large number of facets but relates to cultural (e.g. recreational, aesthetic), health and basic material for a good life (Millennium Ecosystem 2005).

^5^Good soil management will support better carbon sequestration; however, when it comes to different practices of reduced tillage, there may be different trade-offs, depending on the system. Therefore, we need a better understanding of the overall climate trade-offs and biodiversity trade-offs of such management strategies (Garré et al. 2022).

^6^Agroforestry has great potential to support water conservation and supply; as trees can occupy different ecological niches in comparison to annual crops, they also help to reduce surface runoff and to lower evapotranspiration among other things (Scheid et al. 2023).

^7^There could be some potential trade-offs between potential for increased soil organic carbon and N_2_O emissions. Therefore, the net benefits would need to be determined.

Sources for associated co-benefits: Fertilisers: (Jones et al. 2014; Köninger et al. 2021; Morais et al. 2021). *Energy and Fuels:* (Graham et al. 2021) Farm infrastructure: (Millennium Ecosystem 2005) *Tree-related landscape features: (*Fagerholm et al. 2016*). Soil management: (*Pronk et al. 2015; Bijttebier et al. 2018; D’Hose et al. 2018; Hijbeek et al. 2019*). Animal management: (*RDA 2020*) Crops: (*Garré et al. 2022*) Animal products: (*Corscadden et al. 2014*) Animal wastes: (*O'Keeffe et al. 2019*)* Benefits in general (Scheid et al. 2023)

### Aspects to monitor (AtM)

The key performance criteria for CA of Erisman and Verhoeven (2019) broadly complement the strategies of Velasco-Muñoz et al. (2021) and can be used to define aspects that need to be monitored to assess on-farm CA performance and potential. The key performance criteria for CA include, for example, management of nutrient loops, reducing GHG and ammonia, production of renewable energies and water use. These can relate to narrowing resource loops or closing resource loops, depending on activities being implemented. However, they expand the focus wider and come from the premise of “nature-inclusive” farming,^6^ incorporating elements in their framework to be in line with agricultural biodiversity monitoring and regenerative strategies (Erisman et al. 2016; Erisman and Verhoeven 2019). These include aspects relevant for regenerative practices such as: soil quality (e.g. soil organic matter, structure, biology), biodiversity (e.g. crop diversity, land cover types, bird species) and water quality (e.g. no leaching or runoff from the farm). Within their framework of key performance indicators (KPIs) they also incorporate aspects of animal health and welfare (e.g. access to outside, comfortable housing). This is an important feature, which quite often is forgotten in circularity approaches, as good animal welfare practices should be a fundamental practice of any form of agriculture (RDA 2020). In particular for CA, livestock are seen to play a fundamental role in recirculating nutrients, and thus, healthy animals are crucial to fulfil this role (de Boer and van Ittersum 2018; Van Zanten et al. 2019; RDA 2020). Furthermore, within this set of KPIs are aspects which relate to the social capital of the farm, such as the farm’s contribution to the vitality of the local economy (e.g. direct selling, helping neighbours, care farms). These enable the farmers to strengthen their social capital and, through that, their socio-economic position in the food chain and the locality (Erisman and Verhoeven 2019; Bianchi et al. 2020). These social aspects are important to highlight and help the farmer to be aware of, as they may incentivise and encourage the farmer in transitioning their farm.

### Processes and flows relevant for CA at farm level

**Fig. 3:**
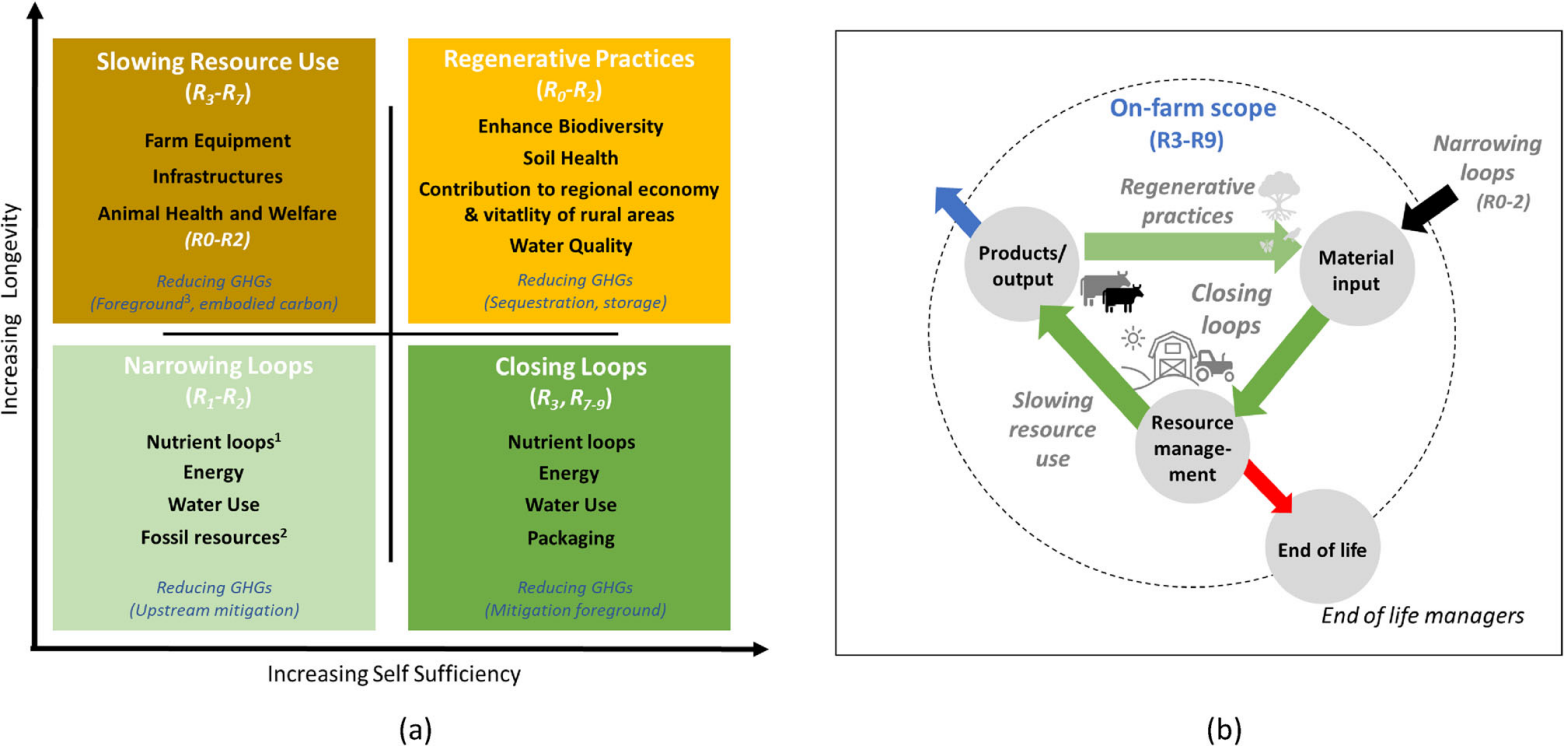
Graphical representation of the: **a** on-farm CA conceptual framework, integrating the strategies of Velasco-Muñoz et al. (2021) with the 9R strategies of Potting et al. (2017) (white text and R letters top) with the key aspects to monitor (AtM) of Erisman and Verhoeven (2019) (black text) and with climate strategies (blue text, bottom). The potential for longevity increases along the y-axis, with increases in effort and dependency on place or self-sufficiency increasing along the *x*-axis. **b** The on-farm processes and flows relevant for the CA strategies (see Sect. “Processes and flows relevant for CA at farm level” for more detail description). *[Note: (1) Refers to Nutrient loops, the nutrients which are mostly “short term”, soil carbon is not included here, but under soil health, as it can take decades to accrue. Note 2. Fossil resources, these refer to non-energy fossil inputs, such as plastic or chemical treatments. Note 3. For slowing resource use, foreground emissions refer to the enteric fermentation and manure management associated with keeping livestock.]*

The major processes and flows relevant for CA at the farm level are: material inputs (short, long duration), resource management, products/outputs and end of life (Table 2, Fig. 3b). These process steps are based on life cycle thinking and are associated with each CA practices implemented as part of a CA strategy. The processes and flows provide the orientation and backbone for supporting life cycle thinking and establishing on-farm life cycle inventories which can help to assess the potential circularity of a farm and its direct and indirect contribution to climate change mitigation (O’Keeffe et al. 2016). Understanding these different processes and flows and how they relate to the CA strategies will help in building robust life cycle inventories, facilitating a more quantitative and technical understanding of the potential impacts of on-farm circular activities.

One of the major adaptations of the Elia et al. (2017) framework is that the loop for CA is not fully circular, as farms are by definition not closed circular systems. They are designed to grow and sell food; hence, there will always be flows leaving the farm that will never be recirculated or returned (blue arrow, Fig. 3a). Therefore, there will be a need, after considering circular R0–R1 strategies (Fig. 1), to import some material inputs onto the farm following the narrowing strategy (e.g. non or low embodied fossil inputs such as food wastes as feed) to safeguard the productiveness of the system (black arrow, Fig. 3b) (de Boer and van Ittersum 2018). Using the strategies of closing loops, slowing resource use and/or regenerative practices (green arrows, Fig. 3b) will ensure recirculation and cascading use of resources on the farm, helping to develop a higher level of self-sufficiency. However, there will be some small number of material flows which cannot reach end of life on the farm e.g. old batteries, old grease and old oil being recycled downstream. These flows, indicated by the red arrow, are no longer useful for the farm and are sent for the end-of-life processes to be dealt with outside the scope of the farm.

Setting a clear system boundary at the farm level and its direct spheres of influence is important to consider for any CA accounting scheme, as this provides a concrete scope, allowing us to track potential resource flows (stock-flows) on, through and off the farm (Robinson et al. 2013). The major focus will be in reducing the inputs, narrowing/R2 strategies (black arrow, Fig. 3b) and balancing this through increasing the recirculation and cascading potential of existing resources on the farm (green arrows). Although the spheres of influence (Fig. 2) may weaken with distance from the on-farm processes, farmers can increase potentially weak influence through conscious sourcing of inputs or through establishing close links to consumers. The following questions may help to discern the level of circularity and which strategy is being implemented: Where is my resource coming from? How is the resource being used on the farm? How is it transformed/converted into its constituent streams or parts? What remains on the farm? What is exported? As well as, what is recirculated and what is the original on-farm source of it?

## Integrated circular framework

The developed conceptual framework is a system thinking approach, using life cycle framing to synergise and link circular strategies to potential climate change strategies in an on-farm context. The strategies are organised in terms of their potential of increasing longevity along the y-axis, with increasing dependency on place or self-sufficiency increasing along the x-axis (Fig. 3a). Here, we refer to longevity as life span, durability and potential circulation time (Figge et al. 2018), which increases along the y-axis (going up). While on the x-axis, going from left to right, the activities are increasing in effort of implementation, but ensure greater self-sufficiency. They also relate to the potential of more longer-term strategies of carbon sequestration and storage.

It is also important to note that in the framework presented here, slowing the resource use and regenerative strategies are qualitatively different from the first two strategies of narrowing and closing loop strategies of material and energy. Slowing and regenerative are concerned with the maintenance flows^7^ that preserve the functions of key agricultural fund elements, such as intact soils that maintain fertility, agro-ecosystems that support pollinators, or livestock herds that provide milk, offspring and physical work. González de Molina et al. (2019) also illustrated how fund elements extend beyond the biophysical cycling, to the social (e.g. the broader agrarian population and individual workers have both basic needs to be met in order to provide stable flows of labour), technical (e.g. farming machines that provide services of traction, transport or processing) and organisational (e.g. flows of money and information that coordinate activities).

Narrowing loops is a strategy aimed at reducing the farms dependency on external inputs. In particular to reduce the farm’s dependency on feed, fodder and fossil inputs and the latter, either directly in the form of energy or indirectly in the form of embodied fossil inputs (i.e. fossil inputs included as an ingredients/component of a product). It covers the following aspects to monitor (AtMs: nutrient loops, energy, water use and fossil resources (Fig. 3, Table S1). The focus of this strategy is to use these resources more efficiently and be conscious of the sufficiency of resources brought onto the farm and used in the production cycles (i.e. required amounts, not excess nutrients, energy, etc.). Through reducing the need for importing nutrients onto the farm, or the use of fossil-based energy or other fossil-based products (e.g. pesticides, lubricants) and reducing water extraction, the farm can also result in reducing its associated upstream GHG emissions (e.g. from material producers, Fig. 2).

Closing loop is a strategy closely coupled, but different from the narrowing loop strategy. If external inputs are being reduced (i.e. narrowing strategy), there will be a need to substitute them with what is available on the farm. The closing loop strategy refers primarily to the cascading use of resources on the farm which fundamentally follows the R strategies of reuse (R3), repurpose, recycle and recover (R7-R9). This strategy, in relation to climate change, is an increased control over the emissions directly produced on the farm, providing more opportunities for farmers to mitigate (lower or stop) these emissions from occurring in the first place (e.g. emissions from slurry storage). The aspects to monitor for this strategy include how nutrients are circulated in and around the farm (spheres of influence), how water is captured or recirculated, energy is generated and how packaging can be reused or repurposed (Table 3, Table S2 for more detailed ontological overview).

Slowing resource use is a strategy to increase the longevity of resources on the farm. It differs from closing loops as the aspects to monitor refer to resource inputs that will be utilised for a longer time frame, such as farm infrastructure (e.g. farm buildings, housing, silage pits, silos) or farm equipment (e.g. tractors, sprayers, sensors, batteries). These farm elements should follow the R strategies which aim to extend lifespan, such as reuse, repair, refurbish, remanufacture or repurpose (R3–R7). Furthermore, it also links to increasing the longevity of livestock (Table S3). If the overall health and quality of life of individual animals are supported (e.g. using R0–R2),^8^ then there is the potential to increase production into later life stages and reduce the requirement to replace them. Thus, increasing longevity of livestock is associated with reduced costs for farmers and also a reduction in direct and indirect GHG emissions (Hoffman and Valencak 2020; Ali 2021). While slowing resource use leads to increasing longevity, this is not per se the same as increasing circularity (Figge et al. 2018). It is a means of indirectly reducing the need for virgin raw materials (also linked to narrowing loops strategy) and also reducing the associated GHG emissions (embodied carbon) (World Green Building 2019; Röck et al. 2020).

The final strategy of regenerative practices can be seen as a longer-term strategy which works with nature and the agricultural community living on and around the farm. It is conceptually rooted in the fund-flow concept as outlined above. It is a strategy which relates to the R strategies of refusing (R0) to use fossil resources or other resources irresponsibly and rethinking (R2) how the farm can function more effectively while regenerating nature and the rural communities. This strategy is also strongly coupled with the strategy of closing loops, because if the natural capital (funds) of the farm is enhanced, then there are potentially more resources that can be recirculated (flows). Thus, also increasing the self-sufficiency of the farm. The AtMs for this strategy link closely to the natural capital of the farm, with regenerative strategies being implemented to enhance increased soil health (e.g. increasing the soil organic matter, soil drainage) or increased biodiversity (e.g. hedgerows, agroforestry, riparian strips) (Fig. 3, Table S4). It is also the strategy which recognises the importance of how circularity should contribute to the socio-economic sustainability of the farm, as it tries to capture the important role a farm can play in contributing to the vitality of the local economy. This can be done in many different ways, e.g. selling directly to the local community, using the farm as a space for community meetings, allowing people access to certain areas of the farm for recreational or mental health purposes (i.e. green care). This strategy can also have a long-term benefit on the climate change mitigation potential and climate neutrality of the farm. The key climate change contribution of this circularity strategy is the potential for sequestering carbon in healthier soils and greater flora diversity. Furthermore, it has the potential for mitigating potential upstream emissions through substitution, as the farm may start producing its own products, such as fertilisers, pest control or timber for buildings (Table 3, Table S4). The best way of understanding the proposed conceptual framework and how it works is through a simplified example. We present this in the next section.

## Hypothetical application to a case study

One simple way of operationalising the conceptual framework could be a scoring tool, based on activity indicators. Here we present how the conceptual framework could potentially be applied in a hypothetical manner, assuming that for each strategy and AtM a series of questions are developed. This can be done two ways either from scratch or from adapting existing sustainability tools (e.g. SMART farm tool (Schader et al. 2016)). The idea would be to use the tool developed from the conceptual framework to assess the main circularity strategies being implemented on the farm in order to identify the potential circularity performance, as well as the potential GHG emission mitigation. However, the real goal of the tool would be to provide a structured approach for talking with the farmers and helping them realise where potential improvements could be implemented on their farm to enhance the farms sustainability.

For our hypothetical case study, we pick an extensive dairy farm in the Netherlands, with 50 dairy cows and a few chickens on 35 hectares, located near a nature-protected area. The farmer is also experimenting with an agroforestry patch, where the cows are also allowed to graze during the summer months. An interview is conducted with the farmer to understand some of the farming practices being implemented on the farm. An overview of potential practices organised according to the circular strategies and AtMs is provided in Table S5. In this hypothetical case the farmer’s circularity strategies are then scored in a very simple manner, based on the number of practices they are implementing out of how many potential practices they could be implementing. There are many ways such a scoring system can be designed, but for the purpose of providing a simple example, this discussion is outside the scope of this paper.

**Fig. 4:**
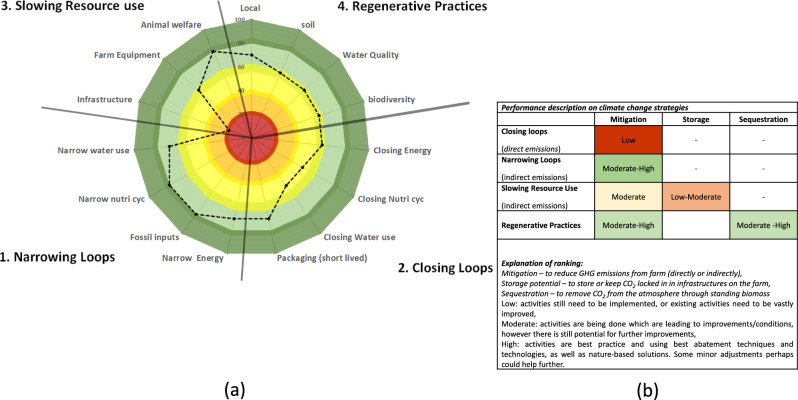
Graphical representation of a hypothetical results dashboard that could be used to communicate with farmers **a** what kind of circular strategies they are using on the farm, **b** where they could make improvements to enhance circularity and climate performance of their farm

The “results” of our hypothetical case can be seen in Fig. 4; we can see the farmer is mostly implementing practices that fall under the narrowing loop strategy. They are also scoring quite high on two of the slowing resource strategies, animal welfare and farm equipment. However, it is clear from Fig. 4 that the farmer needs to work on issues relating to infrastructure due to the low score. Looking into this topic, a crosscutting issue of slurry storage could be discussed with the farmer (e.g. under slow resource use: infrastructures and under closing loops: nutrient cycles). Due to a lack of a sealed slurry pit, it is more than likely that there are large quantities of GHG emissions being released (e.g. CO_2_, NH_3_, N_2_O) (Fig. 4b). This also means that there are potential gaseous losses of nitrogen, thus also reducing the fertilising effect of any manures applied to the grassland (Eggleston et al., 2006; Hergoualc’h et al., 2019). Additionally, these losses may impact the farmer’s yield potential and may reduce the economic viability of his fertilisation plan.

Regarding water management, the farmer appears to be focused on water saving techniques (narrowing loop) and is less focused on capturing or recycling water (closing loops). This is another area for example where the farmer can improve and reduce his water extraction further, thus also potentially creating additional economic gains. The farmer is doing reasonably well on the regenerative strategies too, in particular the soil and pasture management practices that they are implementing, in combination with agroforestry. This allows them to mitigate many direct on-farm GHG emissions, as well as sequester CO_2_ with growing trees (Fig. 4b). These strategies also create synergies by creating ecological niches to improve and enhance the biodiversity on their farm. The best performing regenerative aspect is the farmer’s contribution to the vitality of the local community (e.g. AtM—Local, Fig. 4a). This is done through various activities, such as selling their produce locally, providing a space for the community to meet and being involved in care farming.

With this analysis, another visit could then be paid to the farmer to discuss the results and to help the farmer become more aware of their CA strategies, what is working and where CA practices can be improved or implemented to enhance the overall sustainability of the farm. Additionally, it also helps them to verbalise and describe what they are doing, making it useful to communicate with other farmers or agricultural extension officers, when they are looking for support. While there are many interesting details that could be outlined here, the purpose of this exercise is to show at least one potential option of how the conceptual framework can be operationalised to support farmers in their transition towards CA.

## Discussion

This paper sets out a novel conceptual framework for approaching CA that can ultimately be used to encourage and support farmers to implement more circular practices on their farm. Ultimately, with a more holistic understanding of circular strategies and practices farmers can make more informed decisions and this can contribute to greater climate change mitigation and resilience, as well as enhance the natural and social capital of the farm, thus leading to greater on-farm sustainability. Additionally, at least in the European context, there has not been, to date, such a holistic circularity approach on the farm, linking to the different climate change strategies of mitigation, sequestration and storage. Such an integrated approach will become extremely relevant for the EU to achieve its delegated climate targets. This conceptual framework can help contribute to advancing a more structured, management orientated and coherent discussion on CA and to further identifying if and how current on-farm practices of farmers are circular and how they contribute to on-farm sustainability. While the paper provides concrete examples in a European context, this conceptual framework is also applicable to other regions.

CA is more than technological innovation and according to Grumbine et al. (2021) *“food systems evolve through peoples’ everyday behaviour where the seeds of change are planted that can accumulate and are amplified over time… incremental “small wins”, “small stories”… can be used to inform and inspire*”. Starting at farm scale provides a concrete opportunity for such “small wins”, as it will be farmers and land managers who will implement these CA practices “on the ground” (Silvius et al. 2023). However, it is clear that there cannot be a “one size fits all” solution for implementing CA (Rahmann et al. 2021). There needs to be a greater diversity of solutions and knowledge to fit the diverse needs of farmers. Therefore, with this framework we tried to provide options for all types of farmers to implement more circular strategies, to start with their “small wins”. In this way it can apply to farmers that are taking a more adaptive CA approach (i.e. changing certain aspects of their farm or introducing new technologies—agriculture 4.0 practices^9^) and also to farmers that are taking a more radical CA approach and farming in a totally alternative way to what is currently considered as “conventional” (Dagevos and Lauwere 2021; Sumberg and Giller 2022).

However, while this framework aims to support farmers’ knowledge on circular practices within their sphere of influence, there are many external factors which may also impede their ability or willingness to adopt these circular practices (Mills et al. 2017). In particular policies and legislation are of crucial importance and can either act as a barrier or a driver of farmer CA activities (Crovella et al. 2021; de Lauwere et al. 2022; Happel et al. 2022; López-Serrano et al. 2022; Rótolo et al. 2022). Therefore, adequate policy and legislative support is crucial for the development of CA, with many also calling for a shift from specialisation to a re-mixing and diversification of the farm systems (Schut et al. 2021) at different scales.^10^ Ultimately, polices need to provide opportunities and incentives, while also removing the major barriers which farmers are facing to transition towards CA. Such policies also need to ensure the removal of barriers is also done equitably (Khanna et al. 2024). Such forward thinking policies could play a very important role in supporting the social capital of farmers and the farming community (de Boer and van Ittersum 2018). They could be used to create the mechanisms that encourage and increase the diffusion of on-farm CA interventions through pioneer farmers and farmer-led peer-to-peer learning (Bianchi et al. 2020; de Lauwere et al. 2022), thus creating stronger networks of farmers working in solidarity with one another. In such networks, the use of integrated CA frameworks, as outlined in this paper, could be a useful tool to help verbalise and facilitate this knowledge sharing and diffusion.

When working collaboratively with farmers, the application of the framework to their farm may also help to understand the potential underlying motivations of farmers. For example, when adapting practices towards those found in the regenerative quadrant, where the focus is on ecosystem health, the GHG mitigation becomes the co-benefit, not the focus (Moinet et al. 2023). This is an important focus for many farmers who search for new ways to farm and address both their livelihoods and desire to contribute to the broader well-being of society and nature (Goris et al. 2024). For them, it is about nature first coupled to the health of their farm, then its climate change adaptation or mitigation. It is important to make a distinction here. While climate change and reduction of fossil inputs to the food system are key drivers for policy-level transition to CA, this does not always reflect the intrinsic motivation of farmers to implement CA practices, as we mention above. Facilitating an understanding of these differing drivers, is also a novel aspect of the framework.

However, there are some limitations to the framework. There are overlaps and synergies between the different strategies, and it is sometimes not easy to differentiate between the different strategies due to the many interlinkages. Therefore, greater effort needs to be made to improve the classification of CA practices, based on specific contexts. However, despite these overlaps, proposing these broad overarching strategies combined with more specific AtMs can be seen as a useful system thinking tool, to support on-farm decision making and planning, to encourage more circular farm activities and increase farm-level sustainability. Furthermore, there is a need to understand the spheres of influence of the farm and how this knowledge can be used to support farmers in making decisions to manage trade-offs and create greater on-farm circularity and climate neutrality that extends beyond their farm.

Additionally, while it is clear that CA contributes to and is nested within the broader concepts of sustainability, currently, there are no on-farm tools for measuring the broader concept of circularity presented in this paper, nor how this relates to issues of on-farm sustainability. Thus, farm-level models and tools need to be designed to measure circularity and to determine how CA measures can help achieve more sustainable agricultural practices, as well as potential trade-offs. Therefore, while the framework presented here is still in its infancy, derived from different literature sources, in a next step, the authors would like to develop it further, connecting it with on-farm sustainability tools. Building off existing tools for data collection has many advantages, as it is building from the previous scientific rigour invested in developing them. However, there are also disadvantages, in relation to the inclusion of relevant circularity indicators and potentially incompatible weighting systems. The latter could make it more difficult to understand better the trade-offs and synergies associated with various on-farm circular practices. Therefore, this will require some effort to test and pilot that such a tool is working well. Making a comparative analysis with farmers with different socio-economic, political and biophysical contexts would be useful to gain greater insights into usefulness of the framework and the selected tool. Developing such tools to measure circularity will be essential for the future advancement of CA and the climate neutrality of agricultural systems (Rocchi et al. 2021; Dagevos and Lauwere 2021). The conceptual framework presented in this paper is a first step in this direction, as it can help to support concrete discussions with farmers and to determine how circular their farms are in practice. Furthermore, it will help identify what strategies they have a preference for and what needs to be done to enhance their farms circularity and how this can contribute to the overall on-farm sustainability.

## Conclusion

The scope of CA must be expanded. It needs to be greater than only technical solutions, as any CA practices implemented need to align with sustainability goals, tackle climate change and ensure nature restoration. Therefore, to help support farmers in their transition to more circular, sustainable and climate neutral practices on their farm, a novel framework to encourage farmers to implement more circular practices has been developed and outlined in this paper. The framework has the potential to be used as a thinking tool to support collaborative discussion and dialogue with farmers that can help to broaden their perspectives in relation to CA activities and the related management opportunities on their farms. However, forward thinking policies and instruments are crucial for the development of CA, playing an important role in supporting the social capital of farmers and the farming community. Such policy instruments could be used to create the mechanisms and narratives that encourage and increase the diffusion of on-farm CA interventions through, for example, pioneer farmers and farmer-led peer-to-peer learning, thus creating stronger networks of farmers working in solidarity with one another. In such networks, the use of integrated CA frameworks, such as the one outlined in this paper, could be a useful tool to help verbalise and facilitate this knowledge generation, sharing, implementation and diffusion. Additionally, while climate change and reduction of fossil inputs to the food system are key drivers for policy-level transition to CA, this does not always reflect the intrinsic motivations of farmers to implement CA practices. Facilitating an understanding of these differing drivers, is also a novel aspect of the CA framework presented here.

The framework and discussion presented in this paper is the first attempt to integrate various circular and climate mitigation strategies at the on-farm level, therefore making it novel and useful as a thinking tool, contributing to advancing the discussion on CA and its contribution to sustainable agricultural systems. While the paper provides concrete examples in a European context, this conceptual framework is also applicable to other regions. In a next step, the authors would like to further develop and operationalise the tool in combination with existing on-farm sustainability tools. Developing such tools to measure, evaluate and reflect about circularity will be essential for the future advancement of CA, as well as the climate neutrality and sustainability of agricultural systems.

## Supplementary Information

Below is the link to the electronic supplementary material.

Supplementary file1 (PDF 738 KB)

## Data Availability

All data supporting the conceptual framework outlined in this study are freely available and can be found in the reference list provided at the end of this article and in the supplementary materials.

## Footnotes

1 Nationally determined contributions (NDCs) are efforts by each country to reduce their national GHG emissions and to adapt to the impacts of climate change. The Paris Agreement requests each country to outline and communicate their post-2020 climate actions, known as their NDCs. Together, these climate actions determine whether the world achieves the long-term goals of the Paris Agreement and to reach global peaking of greenhouse gas (GHG) emissions.

2 These policies align with the EU policies on *Land Use and Land Use Change and Forestry*, which establishes an EU-wide net sink target for 2030 with the aim of sequestering 310 Mt CO_2_ within the land-based sectors of the EU by 2030 (European Commission 2018).

3 Nature Restoration Law (NRL), which establishes targets to restore degraded land in the EU, emphasises that the restoration of degraded ecosystems, particularly of habitats with a large potential to capture and store carbon (European Commission 2022).

4 Greenhouse gases (GHG) all behave differently once in the atmosphere, with varying degrees of impact. GHG targets should aim to first prevent emissions from happening. This is known as GHG mitigation and refers to all activities and measures to prevent GHG emissions being released in the first place to the biosphere or atmosphere (Minx et al. 2017; Fuss et al. 2018), whereas carbon sequestration is the process of removing from the atmosphere the CO_2_ that has already been released, sequestering and storing it in geologic formations, the ocean, soil or living things. Storage as biogenic carbon is another form of sequestration, but this refers to carbon stored in harvested biomass (no longer growing) for a given (temporary) period of time depending on the biomass e.g. food is short lived, timber long lived (100 or more years). These are delayed emissions (Garcia-Chavez et al. 2022).

5 These relate to the carbon farming and biobased economy strategies, with woody species being important aspect for agroforestry.

6 An increasingly common Dutch term and concept.

7 The “flow-fund” model developed by Georgescu-Roegen (1971) differentiates between flow elements, consisting of materials and energy that are used up or dissipated during production and consumption activities, and fund elements, consisting of emergent, dissipative structures that both transform flows of resources into more complex forms (e.g. a forest generates stocks and flows of wood and non-timber products) and also maintain key functions that are dependent on an intact “whole” (e.g. a forest may also regulate the climate and harbour complex ecosystems). Because fund elements are dissipative, they require maintenance flows to preserve their functions, i.e. that regenerate and reproduce the fund element. With overuse, fund elements will degrade in quality and exhibit threshold effects where key functions decline rapidly beyond some tipping point of degradation (e.g. the amazon rainforest is believed to be close to an irreversible shift to a savanna ecosystem due to cumulative anthropogenic pressures). The flow-fund model has been adopted for sustainability assessment by Giampietro et al. (2014) to deal with the nexus between agriculture and its biophysical base (land, water and energy). For a recent example applied to the European food system, see Cadillo-Benalcazar et al. (2020).

8 Management of livestock to ensure health and welfare and, thus, potentially increase longevity on the farm will use different R strategies to those for increasing longevity of infrastructure or equipment.

9 The fourth industrial revolution and the digital transformation of the agricultural systems, transitioning towards “Agriculture 4.0” promotes the use of automated equipment and robotics and the precision they can provide in farm operations” (Araújo et al. 2021).

10 These can be either direct diversification on a farm scale or between a network of farmers cooperating and sharing resources depending on the different needs of the farmers involved, i.e. within farm or between farms.
