# Supplementary material for: How to square the circle? A conceptual framework synergising strategies for circular agriculture to tackle climate change and enhance overall on-farm sustainability
Source: Ambio. 2025 Apr 10;54(8):1334–52. doi: 10.1007/s13280-025-02154-4 (PMC12214233; doi:10.1007/s13280-025-02154-4)
Supplement: Supplementary file 1 — Supplementary file1 (PDF 738 KB) [file 13280_2025_2154_MOESM1_ESM.pdf]

Ambio

Supplementary Information

This supplementary information has not been peer reviewed

Title: **How to square the circle? A conceptual framework synergising strategies for circular agriculture to tackle climate change and enhance overall on-farm sustainability**.

S1. Descriptions of Circularity strategies and aspects to monitor

Descriptions of CA strategies and aspects to monitor (AtM) are presented in Tables S1-S4 below. Providing a greater understanding of the conceptual framework's ontology.

Table S1. Descriptions of Strategy "Narrowing loops" and associated Aspects to Monitor (AtMs)

**Strategy**	**AtM**	**Goal**	**Examples**
**Narrowing loops**		To reduce the farms dependency on inputs. In particular fossil inputs, either directly in the form of energy or indirectly in the form of embodied fossil (i.e., fossils included as an ingredient/component of a product).	
	Nutrient Loops	Reducing the amount and need for nutrients being brought onto the farm (outside sphere of influence) from external sources either through reducing e.g., chemical fertilisers, imported animal feed or fodder[1].	Reducing chemical fertilisers through precision fertilisation<br><br>Reverse logistics of (feasible) waste streams from a buyer
	Energy	Reducing the amount of energy used on the farm being sourced from fossil fuels. This refers to energy produced from renewables such as, solar, wind, hydro. It does not refer to energy being produced by recycled or reused materials (see closing loops)	Use of solar panels to produce electricity for animal stall lighting
	Water use	Reducing the amount of water extracted (e.g., from reservoirs, wells, surface water) for on farm production	Using Precision or Drip irrigation only extracting water based on crop demands. Water use efficiency.
	Fossil resources	Reducing the number of fossil-based products being used on the farm i.e., those not covered under energy, but products that have embodied fossil in them either as an ingredient or through their energy intensive production (e.g., lubricants, crop protection products)	Using precision spraying to reduce the need for fossil chemical-based pesticides
	Reducing GHGs	*Reducing the upstream GHG emissions associated with the on farm activities*	*All of the above*

1. Imported animal feed /fodder meaning animal feed that has travelled long distances with a high GHG balance.
2. Reverse logistics refer to the reversal in direction (take back loop) of a production chain, usually referring to the returning of waste streams or by-products resulting from an input which originated on the farm e.g. bread wastes or vegetables returned to farmers for composting or as fodder for livestock (Pinotti et al., 2021; Weber et al., 2023).

Table S2. Descriptions of Strategy “Closing loops” and associated Aspects to Monitor (AtMs)

**Strategy**	**AtM**	**Goal**	**Example**
**Closing loops**		To enhance the cascading use of resources on the farm which fundamentally follows the R strategies of reuse (R3), repurpose and recycle (R7-R8). However, the R strategy of Reducing (R2) should also be considered (e.g., using recirculated streams efficiently)	
	Nutrient Loops	To recycle nutrients within the farm or replenishing as well as possible the nutrient cycling in parts of the farm that have had an export of nutrients (e.g., sold agricultural products).<br>These farms have the ability to produce their own fodder or feed for animals. Or where possible use reverse logistics[1] to bring food or other valuable wastes whose origins began on the farm, back to the farm	Using own manures, compost where possible<br><br>Reverse logistics of (feasible) waste streams from a buyer within sphere of influence
	Energy	Repurposing or recycling streams that are considered wastes to be used for energy production that can supply the on-farm activities with heat and electricity. Energy self-sufficiency	Anaerobic digestion of wastes<br><br>Pellet burner from own Willow coppice
	Water use	To use water capturing techniques for rainwater, to also apply a cascading use of water[2] within the farm to use as long as possible	Using dairy wastewater for fertigation of crops (Lombardi et al., 2022)
	Packaging	To repurpose and reuse packaging on the farm for as long as possible before being sent for recycling	Using fertiliser bags for storage<br><br>Old boxes to store items
	Reducing GHGs	*To reduce the (direct) foreground emissions of the farm*	*Animal slurries stored with in airtight sealed covered tank, very little GHG being emitted*

1.Reverse logistics- see foot note 2 in table S1
2.“ Cascading is a sequence of consecutive uses of water for different purposes”, see Morseletto et al. (2022)

Table S3. Descriptions of Strategy “Slowing Resource Use” and associated Aspects to Monitor (AtMs)			
**Strategy**	**Aspect**	**Goal**	**Example**
**Slowing resource Use**		Refers to the longevity of inputs on the farm. In other words, those inputs that will be utilised for a longer time frame, such as farm infrastructure and farm equipment. These usually involve a capital investment.<br>These farm elements should follow the R strategies which aim to extend lifespan, such as reuse, repair, refurbish, remanufacture or repurpose (R3-R7).<br>This strategy also includes animal health and welfare (R0-R1).	
	Animal Health and Welfare[1]	Animal welfare refers to what matters to animals from their point of view, this refers to their emotional states either positive or negative and its duration. Animal health refers to the absences of disease and that the animal is observed with behaviour that is normal and fitting	Access to outdoors<br><br>Clean housing<br><br>Building features that stimulate
	Farm Infrastructure	To keep the structural integrity and functionality of on farm infrastructure intact for as long as feasible – ensuring a longer functional life span (e.g., farm buildings, housing, silage pits, silos)	Repairing or patching a stall roof to prevent from any further damage to the stall
	Farm Equipment	To maintain the use of farm equipment (e.g., tractors, sprayers, sensors, batteries) for a long time without need to replace them too often or unnecessarily due to lack of care	Servicing the tractor after any major campaign to find any necessary repairs
	Reducing GHGs	Reducing greater energy efficiency of buildings and energy consumption. Reducing direct emissions through maintenance of farm equipment and appropriate livestock managements. Additionally, reducing the embodied carbon of products being brought onto the farm.	See above
1. Definition of animal welfare (Bracke et al., 1999) animal health (Ducrot et al., 2011)			

Table S4. Descriptions of Strategy "Regenerative practices" and associated Aspects to Monitor (AtMs)			
**Strategy**	**Aspect**	**Goal**	**Example**
**Regenerative Practices**		All on farm agricultural practices and actions should enhance the natural and social capital of the farming region (e.g., soils, water, habitats). It is a strategy which is fundamentally rooted in the R strategies of refusing (R0) and rethinking (R2) how the farm can function more effectively and nature inclusively. It functions in combination with the other strategies	
	Enhancing biodiversity	To incorporate agricultural practices that promote biodiversity (e.g., pollinators, birds, flora, nature corridors) throughout the farm and the surrounding landscapes, irrespective of the farm system (e.g., arable or livestock)	Pruning hedgerows or harvesting of crops to allow for bird breeding cycles<br><br>Planting native flora species to encourage insects and pollinators
	Enhancing soil health	To incorporate agricultural practices that aim to enhance the soil ecosystem functioning irrespective of the farm system (e.g., arable or livestock)	Reduced tillage practices<br><br>Application of mulch and soil cover practices
	Water quality	To incorporate agricultural practices that ensure good water quality in water bodies adjacent or in proximity to the farm	No spreading of fertilisers before the specified time of year or before a known heavy rainfall event<br><br>Keeping livestock from accessing sensitive water bodies
	Contribution to regional economy & Vitality of rural areas	To give back and be part of the local community which are in the vicinity of the farm. Taking a place-based approach to production and innovations on the farm	Direct selling - building a customer relationship<br><br>Composting or digesting food waste from local verified sources to produce energy [2]
	Reducing GHGs	Potentially contributing to all three climate change strategies. Mitigation through reduced need for fossil inputs, sequestration through reabsorbing and keeping carbon in the soils and living agroforestry systems, storage in the wood-based products and lignin based products produced on the farm (and thus also contributing back to mitigation through substitution of products)	See above

1. Referring to practices such as those outlined in the soil monitoring law: (https://www.consilium.europa.eu/en/press/press-releases/2024/06/17/soil-monitoring-law-eu-on-the-pathway-to-healthy-soils-by-2050/)
2. Ideally the heat or electricity could be used on the farm (small scale digester), or if it is a cooperative of farmers running the digester with an ample installed capacity, it could be further distributed to the local community through small scale district heating networks.

S2. Application of conceptual framework to a hypothetical case study

Table S5. Summary of on-farm CA assessment based on hypothetical activity indicators		
**CA strategy**	**AtM**	**Activity indicators**
**Narrowing Loops**		
	Fossil inputs	No herbicides, insecticides, fungicides, growth regulators, No reseeding of pastures – good pasture management
	Nutrient Loop management	No chemical fertilizers, NPK (see closing),<br>Small amount of grain feed bought -regionally supplied.
	Energy	Solar panels for the diary and house, energy saving equipment in dairy parlor
	Water use	No irrigation, water saving equipment in dairy
**Closing Loops**		
	Nutrient Loop management	Most of feed comes from on-farm grass fed.<br>Use of own animal manure. However, slurry pits were opened.
	Energy	Combustion furnace using own wood supply from agroforestry management cutting
	Water use	Use of crop residues on soils (soil moisture)
	Packaging (short lived)	Feed bags reused for storage.<br>Most packaging sent off site for recycling
**Slowing resource use**		
	Animal health and welfare	Clean comfortable housing, access to outdoors, pasture grazing most of year when OK to do so. Hedgerows for shelter/shade
	Farm Infrastructure	Slurry pits were not closed fully and looked in need of repair
	Farm equipment	Tractors and machines serviced every year.<br>Very low replacement rate of equipment
**Regenerative practices**		
	Enhancing biodiversity	Planting native fruit trees in Agroforestry patch<br>Pruning of hedgerows to allow bird breeding cycles<br>Flower strips to support pollinators
	Enhancing soil health	Mulching from agroforestry<br>Spreading of manures<br>No traffic (machines or animals) on wet soils
	Water quality	Livestock not able to access river near site<br>Manures spread at a good time of year (no run off , leaching). Water from milking parlor sent to treatment plant (off site)
	Contribution to regional economy & Vitality of the rural areas	Direct on-farm selling of milk/eggs<br>Provide a space for community meetings<br>Also active in care farm initiative
1. Assumed that the cattle are over wintering in pen, with spreading of animal wastes on areas during relevant periods to enhance crop growth and avoid pollution.		

References

Bracke, M.B.M., B.M. Spruijt, J.H.M. Metz. 1999. Overall animal welfare reviewed. Part 3: welfare assessment based on needs and supported by expert opinion. *Wageningen Journal of Life Sciences* 47, https://doi.org/10.18174/njas.v47i3.468

Ducrot, C., B Bed'Hom, V. Béringue, J.B. Coulon, C. Fourichon, J.L. Guérin, S. Krebs, P. Rainard, et al. 2011. Issues and special features of animal health research. *Veterinary Research* 42: 96, https://doi.org/10.1186/1297-9716-42-96.

FAO. 2014. Food and Agriculutral Organisation of the United National. SAFA. Sustainability assessment of food and agricultural systems. Guidelines Version 3.0. Available at:https://www.fao.org/nr/sustainability/sustainability-assessments-safa/en/. Accessed: Feb2020.

Lombardi, B., L. Orden, P. Varela, M. Garay, G.A. Iocoli, A. Montenegro, J. Sáez-Tovar, M. Bustamante, M., et al. 2022. Is Dairy Effluent an Alternative for Maize Crop Fertigation in Semiarid Regions? An Approach to Agronomic and Environmental Effects. *Animals* 12, https://doi.org/10.3390/ani12162025.

Morseletto, P., C.E. Mooren, S. Munaretto. 2022. Circular Economy of Water: Definition, Strategies and Challenges. *Circular Economy and Sustainability* 2: 1463-1477, https://doi.org/10.1007/s43615-022-00165-x.

Pinotti, L., A. Luciano, M. Ottoboni, M. Manoni, L. Ferrari, D. Marchis, M. Tretola. 2021. Recycling food leftovers in feed as opportunity to increase the sustainability of livestock production. *Journal of Cleaner Production* 294: 126290, https://doi.org/10.1016/j.jclepro.2021.126290.

Weber, L., L. Bartek, P. Brancoli, A. Sjölund, M. Eriksson. 2023. Climate change impact of food distribution: The case of reverse logistics for bread in Sweden. *Sustainable Production and Consumption* 36: 386-396, https://doi.org/10.1016/j.spc.2023.01.018.
